# Analysis of the Functional Outcome of Surgically Operated Floating Knee Injury

**DOI:** 10.7759/cureus.105533

**Published:** 2026-03-19

**Authors:** Basava Kiran J K, Ashoka Rakshith, B G Sagar, Tejas J, Mohammed Nafras A A, Prajwal L Gowda

**Affiliations:** 1 Orthopedics and Trauma, Adichunchanagiri Institute of Medical Sciences, Adichunchanagiri University, Mandya, IND; 2 Orthopedics, Adichunchanagiri Institute of Medical Sciences, Adichunchanagiri University, Mandya, IND

**Keywords:** floating knee injuries, functional outcome, ipsilateral femur and tibia fracture, karlström and olerud criteria, modified fraser’s classification, surgical fixation

## Abstract

Background and objective

A simultaneous ipsilateral femur and tibia fracture that separates the knee from the rest of the limb is known as a "floating knee." Both extra-articular and intra-articular fractures are included. The aim of this study is to analyze the functional outcome in surgically operated floating knee injuries.

Methods

The prospective study was conducted with 20 patients with floating knee injuries who were operated on at the Adichunchanagiri Institute of Medical Sciences. These fractures are classified by using the modified Fraser's classification. Patients were followed up for a minimum of six months. The clinical and functional outcome of these patients was assessed by using the Karlström and Olerud criteria.

Results

The mean age of the patients was 34.50±13.48 years. The mechanism of injury was road traffic accidents (RTA) in 19 patients and a fall from height in one patient. Based on Fraser's classification, 11 had Type I fracture, two had Type IIA fracture, one had Type IIB fracture, and six had Type IIC fracture (p=0.34). Based on treatment, three patients with femur fractures were treated with cannulated cancellous (CC) screws. According to the Karlström and Olerud criteria, the outcome was acceptable in four patients (20%), excellent in seven patients (35%), good in six patients (30%), and poor in three patients (15%). According to Fraser's classification, seven patients with Type I fractures (63.6%) had excellent outcomes.

Conclusion

Long-term rehabilitation and careful surgical planning are advised for the fixation of the fractures. The final result for these individuals is determined by a combination of these factors.

## Introduction

A simultaneous ipsilateral femur and tibia fracture that separates the knee from the rest of the limb is known as a "floating knee" [1]. Both extra-articular and intra-articular fractures are included [2]. Growing industrialization has led to an increase in the prevalence of floating knee injuries [3].

Most cases result from road traffic accidents (RTA), followed by falls from height. The term "floating knee" was first introduced by McBryde in 1965 to describe simultaneous fractures of the ipsilateral femur and tibia [4]. These high-energy injuries, often part of polytrauma, involve severe bone and soft tissue damage and are frequently associated with life-threatening head, chest, or abdominal injuries.

Infection, severe blood loss, fat embolism, delayed union or nonunion, knee stiffness, extended hospitalization, and incapacity to support weight are some additional consequences linked to floating knee injuries [5]. It is not unexpected that these injuries are linked to other injuries (soft tissue and bony) because it takes tremendous power to break two of the strongest bones in the body [6].

A floating knee is not a frequent injury; however, its precise prevalence is unclear. Over an 11-year period, 222 instances comprised the biggest series of patients recorded in the literature. There are several approaches to managing them, and they may also be linked to potentially fatal trauma [7].

In addition to ipsilateral femur and tibia fractures, the floating knee is a complicated injury. Injuries and the kind of fracture (open, intra-articular, or comminution) are predictive factors for both the initial and end outcomes of the patients [8]. Given this background, the present study aims to evaluate the functional outcomes of surgically treated floating knee injuries at Adichunchanagiri Institute of Medical Sciences.

## Materials and methods

This hospital-based prospective study was conducted in the Department of Orthopedics at Adichunchanagiri Hospital and Medical Research Centre, B.G. Nagar, Mandya. The study involved patients who presented to the Orthopedics Outpatient Department or the Emergency Department with a diagnosis of floating knee injury. It was carried out from February 2023 to December 2024, with a total sample size of 20 cases. The Institutional Ethics Committee of Adichunchanagiri Institute of Medical Sciences issued approval AIMS/IEC/109/2023. The aim was to evaluate the functional outcome of surgically treated floating knee injuries. A formal sample size calculation was not performed due to the relatively rare incidence of floating knee injuries. The study included all consecutive eligible cases presenting during the study period. Therefore, the sample size represents a convenience sample. The study may not have been adequately powered to detect small differences in subgroup analyses, and the results should be interpreted cautiously.

The data for this study were obtained from patients with floating knee injuries at the Adichunchanagiri Institute of Medical Sciences. All types classified under the modified Fraser's classification were included and managed surgically using various fixation methods. Cases involving ipsilateral femur and tibia fractures extending into the hip or ankle joints, as well as pathological fractures, were excluded. Patients lost to follow-up or with a follow-up period of less than four months were also excluded from the analysis. Functional outcomes were evaluated after bony union using the Karlström criteria [9].

Procedures

Surgical options for managing floating knee injuries include external fixation, intramedullary nailing, ligament repair, and open reduction and internal fixation (ORIF) with plates and screws. The choice of surgical approach depends on several factors, including the patient's age, the surgeon's expertise, the condition of the soft tissues, the specific fracture pattern, and the presence of any associated injuries [10]. In most cases, femoral fixation was performed first to restore limb alignment and facilitate stabilization. However, the sequence was individualized based on patient hemodynamic stability, soft tissue condition, fracture configuration, and associated injuries. In cases of severe open tibial fractures or compromised soft tissues, tibial stabilization was prioritized. No rigid institutional protocol mandated a fixed sequence, and decisions were made according to damage-control orthopedic principles when required.

Statistical analysis

Data were entered into Microsoft Excel 2016 (Microsoft Corp., Redmond, WA) and analyzed using SPSS version 28 (IBM Corp., Armonk, NY). Descriptive statistics were used to summarize data. For categorical variables, Fisher's exact test was applied when expected cell counts were less than five. The chi-square test was used, where assumptions were met. Given the exploratory nature of subgroup analyses and the small sample size, no adjustment for multiple comparisons was performed. A p-value of <0.05 was considered statistically significant; however, findings should be interpreted cautiously.

## Results

Based on the nature of the fractures, 18 patients (90%) had closed femur fractures, while 11 patients (55%) had closed tibia fractures. Among the open fractures, one patient (5%) had a Grade I tibia fracture, and another (5%) had a Grade II femur fracture. Additionally, two patients (10%) sustained Grade II tibia fractures, three patients (15%) had Grade IIIA tibia fractures, and Grade IIIB fractures were observed in four patients (20%) (Table 1).

**Table 1:** Mean Age Distribution of the Subjects

Variable	N	Minimum	Maximum	Mean	SD
Age (years)	20	19.0	66.0	34.50	13.48

According to the Karlström and Olerud criteria, outcomes were excellent in seven (35%), good in six (30%), acceptable in four (20%), and poor in three (15%) patients (Table 2).

**Table 2:** Distribution of the Subjects Based on Functional Outcome (the Karlström and Olerud Criteria) A p-value of <0.05 was considered statistically significant df: degrees of freedom

Outcome	Frequency	Percent (%)
Acceptable	4	20.0
Excellent	7	35.0
Good	6	30.0
Poor	3	15.0
Total	20	100.0
Chi-square (χ²)	2.80	
df	3	
P-value	0.423	
Effect size (Cramer's V)	0.26	

In the present study, seven patients (35%) with Fraser's Type I fractures had excellent outcomes, and three Type I, one Type IIA, and one Type IIC fractures had good outcomes, while one Type IIA, one Type IIB, and two Type IIC had acceptable outcomes. Poor outcomes were seen in one Type I and three Type IIC fractures (Table 3).

**Table 3:** Outcome Based on Fraser's Type A p-value of <0.05 was considered statistically significant df: degrees of freedom

Functional Outcome	Type I	Type IIA	Type IIB	Type IIC	Total	Percentage (%)
Excellent	7	0	0	0	7	35
Good	3	1	0	1	5	25
Acceptable	0	1	1	2	4	20
Poor	1	0	0	3	4	20
Total	11	2	1	6	20	100
Chi-square (χ²)	15.74					
df	9					
P-value	0.073					
Effect size (Cramer's V)	0.63					

Outcomes were further analyzed based on the type of fracture. Among femur fractures, excellent results were predominantly seen in closed injuries, while poor outcomes were observed in Grade II and Grade IIIB fractures. In tibia fractures, Grade I and closed injuries yielded excellent results, whereas Grade IIIB fractures were associated with poor outcomes (Table 4).

**Table 4:** Outcome Based on the Type of Fracture A p-value of <0.05 was considered statistically significant df, degree of freedom; G, grade

Outcome	Femur, Closed	G I	G II	G IIIA	G IIIB	Total (Femur)	Tibia, Closed	G I	G II	G IIIA	G IIIB	Total (Tibia)
Excellent	8	0	0	0	0	8	7	1	0	0	0	8
Good	6	0	0	0	0	6	3	0	1	2	0	6
Acceptable	4	0	0	0	0	4	1	0	1	1	1	4
Poor	0	0	1	0	1	2	0	0	1	0	1	2
Chi-square (χ²)	19.56											
df	12											
P-value	0.075											
Effect size (Cramer's V)	0.49											

When assessed by fracture pattern, extra-articular fractures demonstrated superior results compared to intra-articular fractures, which were associated with poorer functional outcomes (Table 5).

**Table 5:** Outcome Based on the Pattern of Fracture A p-value of <0.05 was considered statistically significant df: degrees of freedom

Outcome	Femur, Intra	Femur, Extra	Tibia, Intra	Tibia, Extra
Excellent	0	7	0	7
Good	1	5	2	4
Acceptable	3	1	3	1
Poor	3	0	3	0
Chi-square (χ²)	13.02			
df	3			
P-value	0.004			
Effect size (Cramer's V)	0.57			

The analysis of outcomes based on treatment modality showed that both intramedullary nailings (femur+tibia) yielded the best results, with 87.5% excellent and 12.5% good outcomes, whereas double plating was associated with poor prognosis (Table 6). Treatment allocation in this study was not randomized and depended on fracture configuration, soft tissue condition, and surgeon's discretion. Therefore, the observed better outcomes with intramedullary nailing may reflect less severe injury patterns rather than the superiority of the implant itself. These findings should be interpreted as observational associations rather than causal conclusions.

**Table 6:** Outcome Based on Treatment Modality A p-value of <0.05 was considered statistically significant F, femur; T, tibia; IM, intramedullary; CC, cannulated cancellous; df, degrees of freedom

Implant Used	Cases	Excellent	Good	Acceptable	Poor
Both IM nailing (F+T)	8	7	1	0	0
Both plating (F+T)	4	0	0	1	3
Nailing (F)+plating (T)	3	0	2	1	0
Plating (F)+nailing (T)	1	0	1	0	0
IM nail (F)+external fixator (T)	1	0	1	0	0
CC screw (F)+plate (T)	3	0	1	2	0
Total	20	7	6	4	3
Chi-square (χ²)	25.91				
df	15				
P-value	0.039				
Effect size (Cramer's V)	0.82				

**Figure 1:**
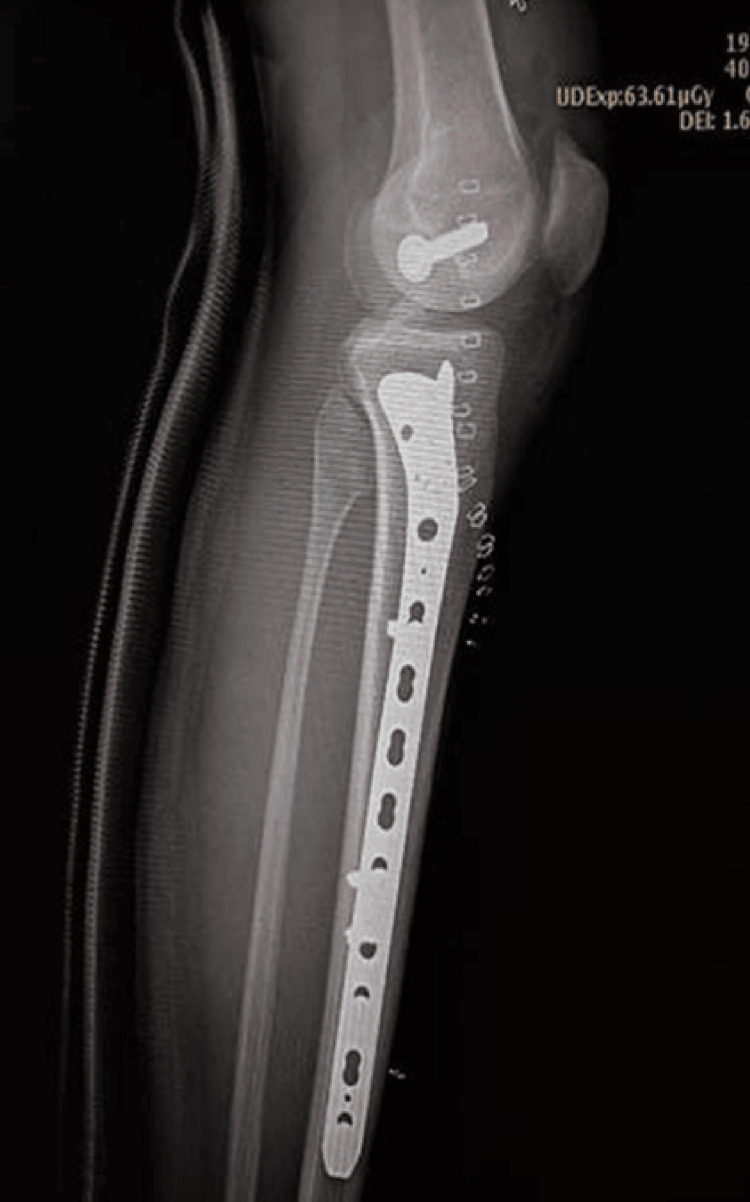
Immediate Postoperative Radiograph of the Left Lower Limb Showing Fraser Type II-C Floating Knee Injury (Closed Type) Following Surgical Fixation.

Figure 1 presents a pre-operative radiograph showing Fraser's Type I floating knee injury.

**Figure 2:**
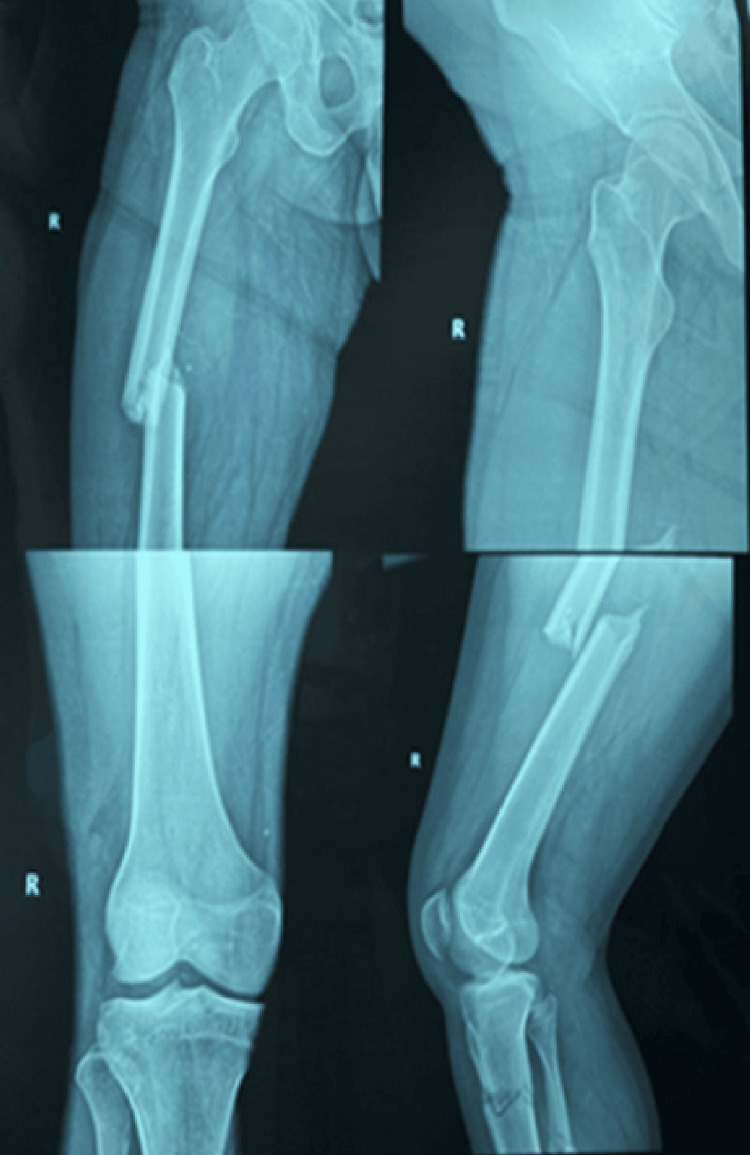
Pre-operative Radiograph Demonstrating Fraser Type I Floating Knee Injury Prior to Surgical Intervention.

Figure 2 presents a postoperative radiograph of the same case at 10 months of follow-up, demonstrating complete union.

**Figure 3:**
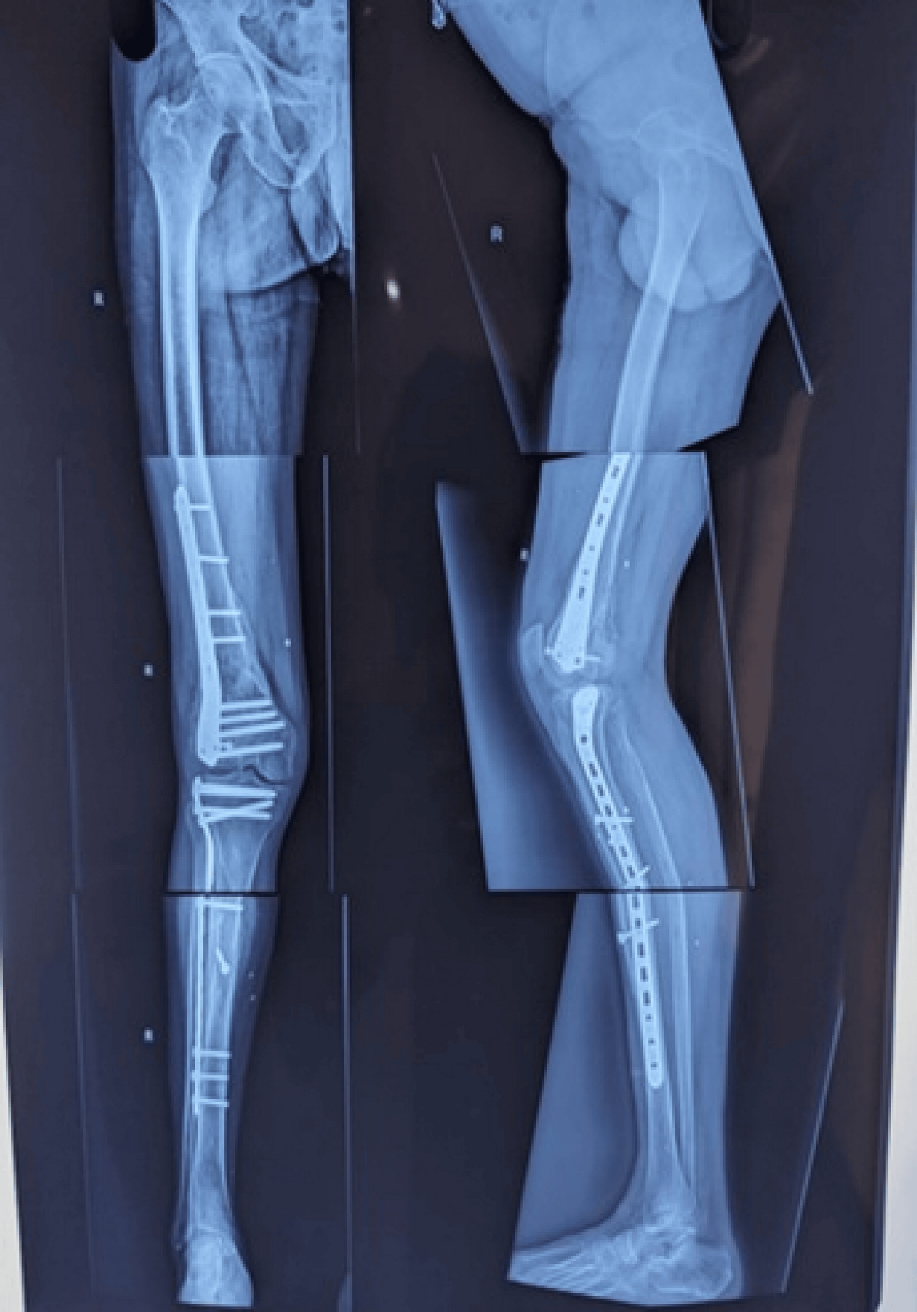
One-Year Postoperative Follow-Up Radiograph of Fraser Type II-C Floating Knee Injury (Closed Type), Showing Satisfactory Alignment and Evidence of Fracture Healing.

Figure 3 presents a clinical photograph showing the patient performing squatting at the final follow-up, demonstrating excellent functional recovery.

## Discussion

Floating knee injuries often involve extensive soft tissue damage and are frequently associated with serious complications such as thoracic and abdominal (visceral) injuries, spinal cord trauma, and potentially life-threatening brain injuries [11]. The early surgical stabilization of femur and tibia fractures, along with timely rehabilitation, is essential for attaining optimal clinical and functional results [12,13]. Timely rehabilitation further contributes to favorable outcomes by minimizing joint stiffness and promoting early return to activities of daily living [14]. Treatment planning should focus on addressing each fracture in the affected limb to maximize the chances of a good or excellent recovery. However, these decisions must be carefully considered within the broader context of the patient's overall health and the severity of their injuries [15]. This study aims to evaluate the functional outcomes following the surgical management of floating knee injuries.

In the present study, the mean age of the patients was 34.50±13.48 years (p=0.34). Similarly, another study by Shukla et al. noted that the mean age was 40.83 years [10]. On the contrary, another study by Rethnam et al. reported the average age as 28 years [7]. Patel et al. observed that 35.5% of the patients belonged to the 20-30-year age group [11].

In the present study, 11 (55%) had Type I fracture, two (10%) had Type IIA fracture, one (5%) had Type IIB fracture, and six (30%) had Type IIC fracture. Similarly, Yadav et al. noted that there were five Type I, two Type IIA, two Type IIB, and three Type IIC floating knee injuries according to Fraser's classification [12]. Patel et al. found that the most common injury type was Fraser's Type I (48.93%), followed by Type IIA (17.02%), Type IIB (14.89%), and Type IIC (19.14%) [11]. These findings concur with those of Shukla et al., who reported 15 (50.0%) fractures of Fraser's Type I, four (13.3%) Type IIA, five (16.7%) Type IIB, and six (20.0%) Type IIC, with the majority in Type I, followed by Type IIC [10].

In the present study, according to the Karlström and Olerud criteria, outcomes were acceptable in four patients (20%), excellent in seven (35%), good in six (30%), and poor in three (15%). Based on Fraser's classification, seven patients with Type I fractures (63.6%) had excellent outcomes, four patients with Type I fractures and two (22.2%) with Type II fractures had good outcomes, four (44.4%) with Type II fractures had acceptable outcomes, and three (33.3%) with Type II fractures had poor outcomes. The association between Fraser's classification and functional outcome did not reach statistical significance (p=0.073), although a trend toward better outcomes in Type I fractures was observed. Yadav et al. reported that functional assessment based on the modified Karlström criteria after bony union was excellent in three, good in five, fair in three, and poor in one case [12]. Rethnam et al. found excellent outcomes in 15, good in nine, acceptable in two, and poor in three patients [7].

This study has several limitations. The small sample size and single-center design may limit the generalizability of the findings. Additionally, the minimum follow-up period was six months, meaning that only short-term outcomes were assessed in some cases, potentially influencing the overall results. Undetected ligament injuries may also have affected outcomes, as routine MRI scans were not performed due to financial constraints.

## Conclusions

Floating knee injuries represent complex high-energy trauma requiring individualized surgical planning. In this observational study, better functional outcomes were more commonly seen in extra-articular fractures and in patients managed with intramedullary nailing; however, treatment allocation was not randomized. Therefore, these findings should be interpreted as associations rather than definitive treatment recommendations. Larger multicenter studies with adequate statistical power are required to establish standardized management protocols. The key finding of this study is that fracture pattern (particularly intra-articular involvement) appears to influence functional outcome more strongly than the fixation method itself in surgically managed floating knee injuries. While adjunct imaging and comprehensive ligament assessment may be clinically beneficial, these were not systematically evaluated in the present study and therefore cannot be recommended based on our findings alone.
